# Prevalence of Vitamin D Deficiency and Osteoporosis Among Patients Undergoing Elective Joint Replacement at a High-Volume Centre in Malaysia

**DOI:** 10.7759/cureus.117921

**Published:** 2026-10-14

**Authors:** Rahul Lingam, Jade Pei Yuik Ho, Kunalan Ganthel Annamalai

**Affiliations:** 1 Department of Orthopaedics, Hospital Kuala Lumpur, Kuala Lumpur, MYS

**Keywords:** arthroplasty, bone health optimisation, bone mineral density, osteoporosis, vitamin d deficiency

## Abstract

Background

Bone health optimisation (BHO) is increasingly recognised as an important strategy for improving patient outcomes in orthopaedic surgery. Vitamin D plays a vital role in bone health, but the burden of vitamin D deficiency and osteoporosis among elective joint replacement candidates in Malaysia’s multi-ethnic population remains poorly characterised.

Methods

In this prospective observational cohort study, 204 patients aged ≥50 years who were listed for elective primary or revision arthroplasty at Hospital Kuala Lumpur between May 2025 and April 2026 underwent preoperative assessment of serum vitamin D, calcium, and albumin levels, together with a bone mineral density (BMD) scan.

Results

Vitamin D deficiency (<50 nmol/L) was present in 43.6% (n = 89) of patients, insufficiency (50-74 nmol/L) in 32.8% (n = 67), and sufficiency (≥75 nmol/L) in 23.5% (n = 48). Osteoporosis and osteopenia were present in 28.9% (n = 59) and 59.3% (n = 121) of patients, respectively. Hypoalbuminemia was present in 91 patients (44.6%). Malay patients had a significantly higher prevalence of vitamin D deficiency than Indian and Chinese patients (χ² = 28.559, p < 0.001), while Indian patients had significantly lower albumin levels (χ² = 12.192, p = 0.002).

Conclusion

Vitamin D deficiency, osteoporosis, and malnutrition were highly prevalent among elective arthroplasty candidates in this Malaysian cohort. These findings support the routine incorporation of serum vitamin D assessment and BMD screening into the preoperative workup.

## Introduction

Arthroplasty remains the most effective and satisfactory treatment for advanced osteoarthritis, restoring mobility and improving quality of life. Historically, much research and focus have been directed towards implant survivorship, including implant design, biomaterial durability, and precise component alignment. Currently, the surgical population has broadened to include both physically active younger patients and older patients with multiple systemic comorbidities. Long-term prosthetic survivorship is now recognised to depend as much on the biological quality of the host bone surrounding the implant as on the implant itself [1,2]. This recognition has given rise to the concept of bone health optimisation (BHO), a more holistic framework for improving surgical outcomes, including those of arthroplasty.

The rationale is that the osseous scaffold should be capable of withstanding the substantial immediate and long-term mechanical loads imposed by prosthetic hardware. In cementless arthroplasty, the implant initially depends on mechanical press-fit stability, followed by secondary stability achieved through biological osseointegration. In contrast, cemented implants require adequate cement interdigitation into host bone of sufficient quality. When the recipient bone is osteopenic or osteoporotic, the risks of early aseptic loosening, implant subsidence, and periprosthetic fracture increase accordingly [1,2].

Among the modifiable determinants targeted by BHO, vitamin D plays a pivotal role in bone health, which may subsequently influence surgical outcomes. Classically recognised for its role in calcium homeostasis and bone mineralisation, vitamin D promotes calcium absorption and drives the differentiation of mesenchymal stem cells into osteoblasts [3]. Vitamin D also influences immune function and muscular contraction [2].

Despite this well-established significance, vitamin D deficiency remains highly prevalent among orthopaedic surgical candidates, affecting an estimated 53-75% of patients [2,4]. Circulating vitamin D levels may decrease by up to 40% during the immediate postoperative period [5]. Given that the background prevalence of vitamin D deficiency in the general Malaysian population has been reported to be 64% [6], further population-specific data among orthopaedic surgical candidates are needed to guide the preoperative implementation of BHO protocols. This study therefore aimed to determine the prevalence of vitamin D deficiency and osteoporosis and their interrelationship among multiethnic patients undergoing elective joint replacement at a high-volume Malaysian centre. Additionally, serum calcium and albumin were also assessed due to their relevance to bone metabolism.

## Materials and methods

Study design and patient selection

This prospective observational cohort study was conducted at Hospital Kuala Lumpur, the main national public referral centre in Malaysia, between May 2025 and April 2026. Ethical approval was obtained from the Medical Research and Ethics Committee (NMRR ID-25-00198-IND), and written informed consent was obtained from all participants prior to enrolment. Patients were assessed to document their medical history and current medication use.

Inclusion criteria

Patients were eligible for inclusion if they were aged ≥50 years at the time of screening and were scheduled to undergo elective primary or revision arthroplasty.

Exclusion criteria

Patients were excluded if they had a history of metabolic bone disease other than primary osteoporosis (e.g., Paget's disease, hyperparathyroidism, or renal osteodystrophy), active malignancy or bone metastases, or end-stage renal failure (estimated glomerular filtration rate <15 mL/min/1.73 m²). Patients taking medications that alter bone metabolism, such as glucocorticoids or bisphosphonates, were also excluded.

Sample size calculation

The sample size was calculated using Cochran’s formula [7], with an expected prevalence of 64% (based on a study by Mohd Saffian et al. [6]), a confidence interval of 95%, a margin of error of 0.05, and with a finite population correction (estimated 400 elective surgeries yearly). The minimum sample size required was 189.

Serum biochemical assessment

Peripheral venous blood samples were collected from all patients during the routine preoperative assessment, and additional blood investigations were performed specifically for this study. The following biochemical parameters were measured.

Serum Vitamin D

Serum vitamin D was measured using a chemiluminescent immunoassay (CLIA) (Roche Diagnostics, Mannheim, Germany). Vitamin D status was categorised according to the Endocrine Society Clinical Practice Guidelines as deficient (<50 nmol/L), insufficient (50-74 nmol/L), or sufficient (≥75 nmol/L) [8].

Calcium and Albumin

Total serum calcium (mmol/L) and serum albumin (g/L) were measured using standard laboratory methods. Reference ranges were defined as 2.20-2.55 mmol/L for serum calcium and 35-52 g/L for serum albumin.

Bone mineral density (BMD) assessment

All enrolled patients underwent preoperative dual-energy X-ray absorptiometry (DXA) (Discovery W, Hologic, Marlborough, MA) to assess baseline BMD. Scans were routinely performed at the lumbar spine (L1-L4) and hip. However, in the presence of metal artifacts at either site, the distal one-third of the radius (forearm) was scanned as an alternative. The lowest T-score across all evaluated sites was used in this study. Bone density status was classified according to World Health Organization (WHO) T-score criteria as normal (T-score ≥ -1.0), osteopenia (T-score -1.0 to -2.5), or osteoporosis (T-score ≤ -2.5) [9].

Statistical analysis

Statistical analysis was performed using IBM SPSS Statistics (version 28, IBM Corp., Armonk, NY). Data were tabulated and summarised descriptively. Categorical variables were compared using the chi-square (χ²) test. A p-value of <0.05 was considered statistically significant.

In addition to the primary analyses, exploratory analyses were conducted to further characterise the study population and examine potential associations of age, sex, ethnicity, serum calcium, and serum albumin with vitamin D status and bone health. These analyses were not predefined secondary objectives and were therefore interpreted as exploratory findings.

## Results

A total of 380 patients were screened between May 2025 and April 2026. After excluding those who declined participation or did not fulfil the inclusion and exclusion criteria, 204 patients were enrolled in this study. The cohort comprised 149 women (73.0%) and 55 men (27.0%). By ethnicity, 37.7% (n = 77) were Chinese, 34.8% (n = 71) were Indian, and 27.5% (n = 56) were Malay. Patient age ranged from 52 to 88 years, with the largest proportion aged 70-79 years (42.2%, n = 86), followed by those aged 60-69 years (38.7%, n = 79). Prior to enrolment, all participants were already receiving oral calcium and vitamin D supplements. Hypoalbuminemia was present in 44.6% of patients (n = 91) (Table 1). Osteoporosis was found to be in 28.9% of patients (Table 2).

**Table 1:** Serum vitamin D, calcium, and albumin status by ethnicity.

Ethnicity	Vitamin D (nmol/L)			Calcium (mmol/L)			Albumin (g/L)	
	Deficient (<50)	Insufficient (50-74)	Sufficient (≥75)	Low (<2.20)	Normal (2.20-2.55)	High (>2.55)	Low (<35)	Normal (35-52)
Chinese	21/77 (27.2%)	26/77 (33.8%)	30/77 (39.0%)	9/77 (11.7%)	61/77 (79.2%)	7/77 (9.1%)	25/77 (32.5%)	52/77 (67.5%)
Indian	30/71 (42.3%)	27/71 (38.0%)	14/71 (19.7%)	3/71 (4.3%)	64/71 (90.1%)	4/71 (5.6%)	43/71 (60.6%)	28/71 (39.4%)
Malay	38/56 (67.9%)	14/56 (25.0%)	4/56 (7.1%)	6/56 (10.7%)	48/56 (85.7%)	2/56 (3.6%)	23/56 (41.1%)	33/56 (58.9%)
Total (n, %)	89 (43.6%)	67 (32.9%)	48 (23.5%)	18 (8.8%)	173 (84.8%)	13 (6.4%)	91 (44.6%)	113 (55.4%)

**Table 2:** Bone mineral density (BMD) categories by ethnicity.

Ethnicity	T-score		
	Normal (≥ -1.0)	Osteopenia (-1.0 to -2.5)	Osteoporosis (≤ -2.5)
Chinese	6/77 (7.8%)	46/77 (59.7%)	25/77 (32.5%)
Indian	11/71 (15.5%)	40/71 (56.3%)	20/71 (28.2%)
Malay	7/56 (12.5%)	35/56 (62.5%)	14/56 (25.0%)
Total (n, %)	24 (11.8%)	121 (59.3%)	59 (28.9%)

Chinese patients exhibited a higher prevalence of osteoporosis (Figure 1) than the other ethnic groups, although this difference was not statistically significant (χ² = 2.748, p = 0.601). Malay patients demonstrated a significantly higher prevalence of vitamin D deficiency (Figure 2) than Indian and Chinese patients, indicating a significant association between ethnicity and vitamin D status (χ² = 28.559, p < 0.001). The chi-square test revealed no significant association between ethnicity and calcium levels. Conversely, albumin status varied significantly across ethnic groups (χ² = 12.192, p = 0.002), with Indian patients exhibiting lower albumin levels than their counterparts (Figure 3).

**Figure 1:**
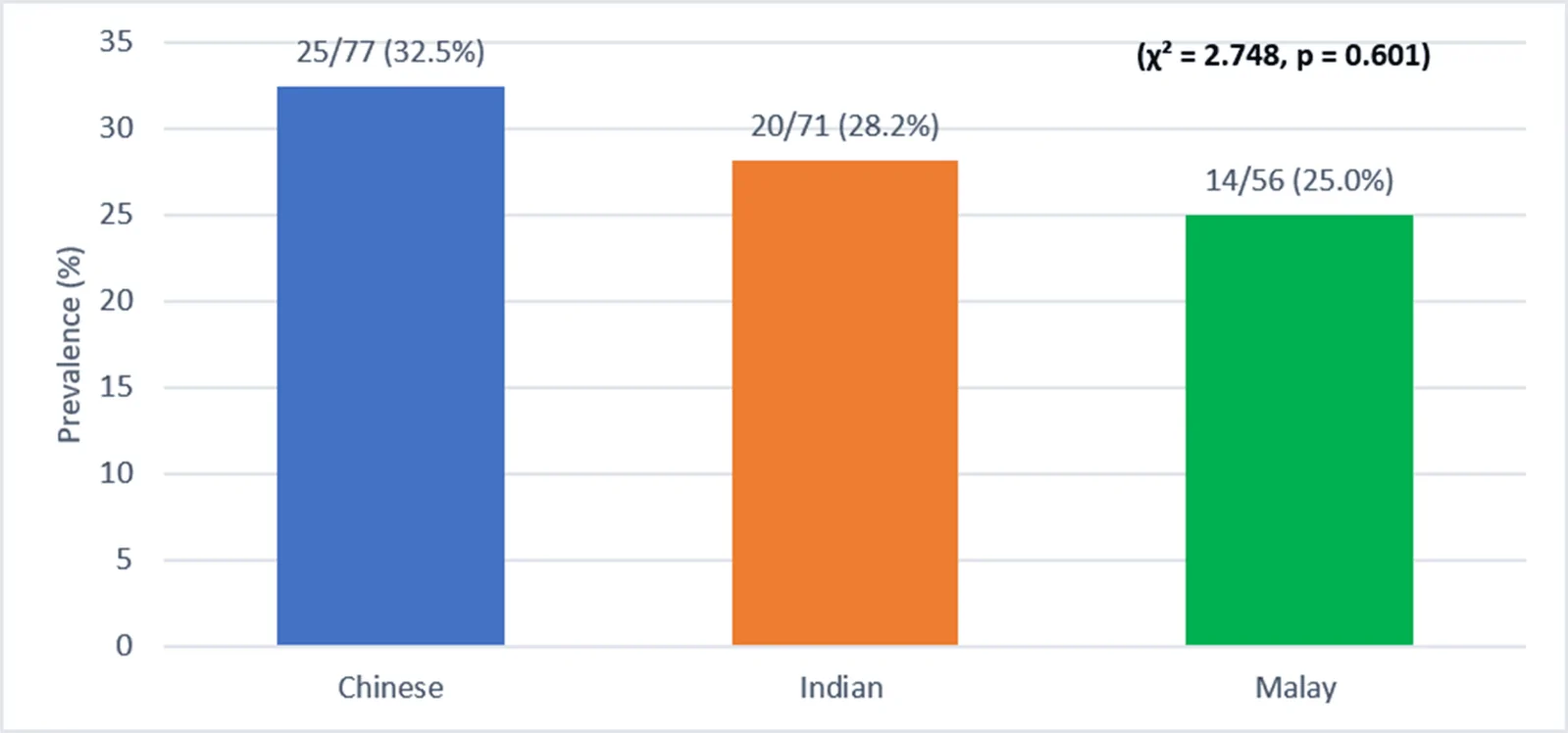
Prevalence of osteoporosis by ethnicity. χ² = chi-square test

**Figure 2:**
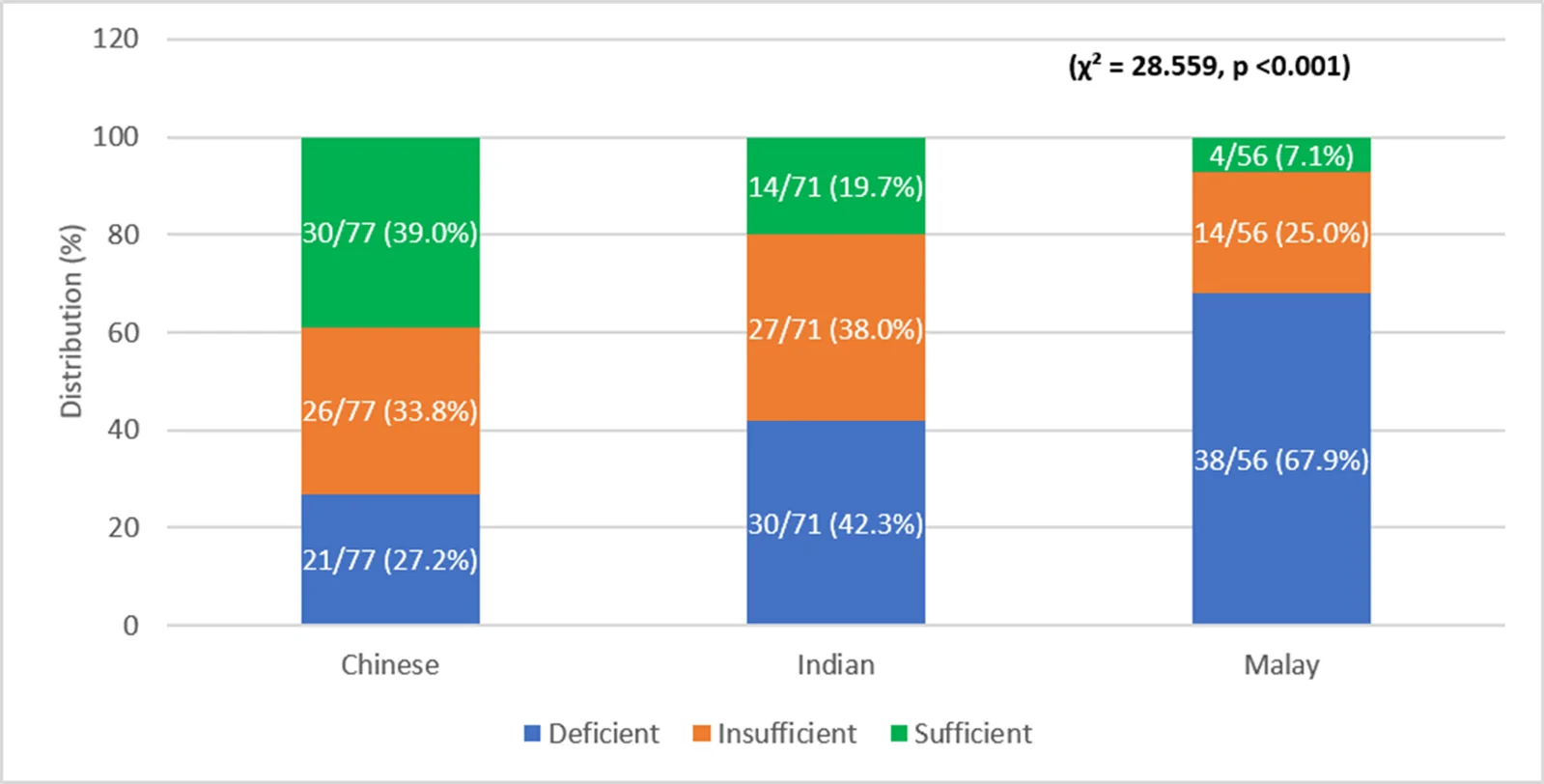
Serum vitamin D levels within each ethnic group. χ² = chi-square test

**Figure 3:**
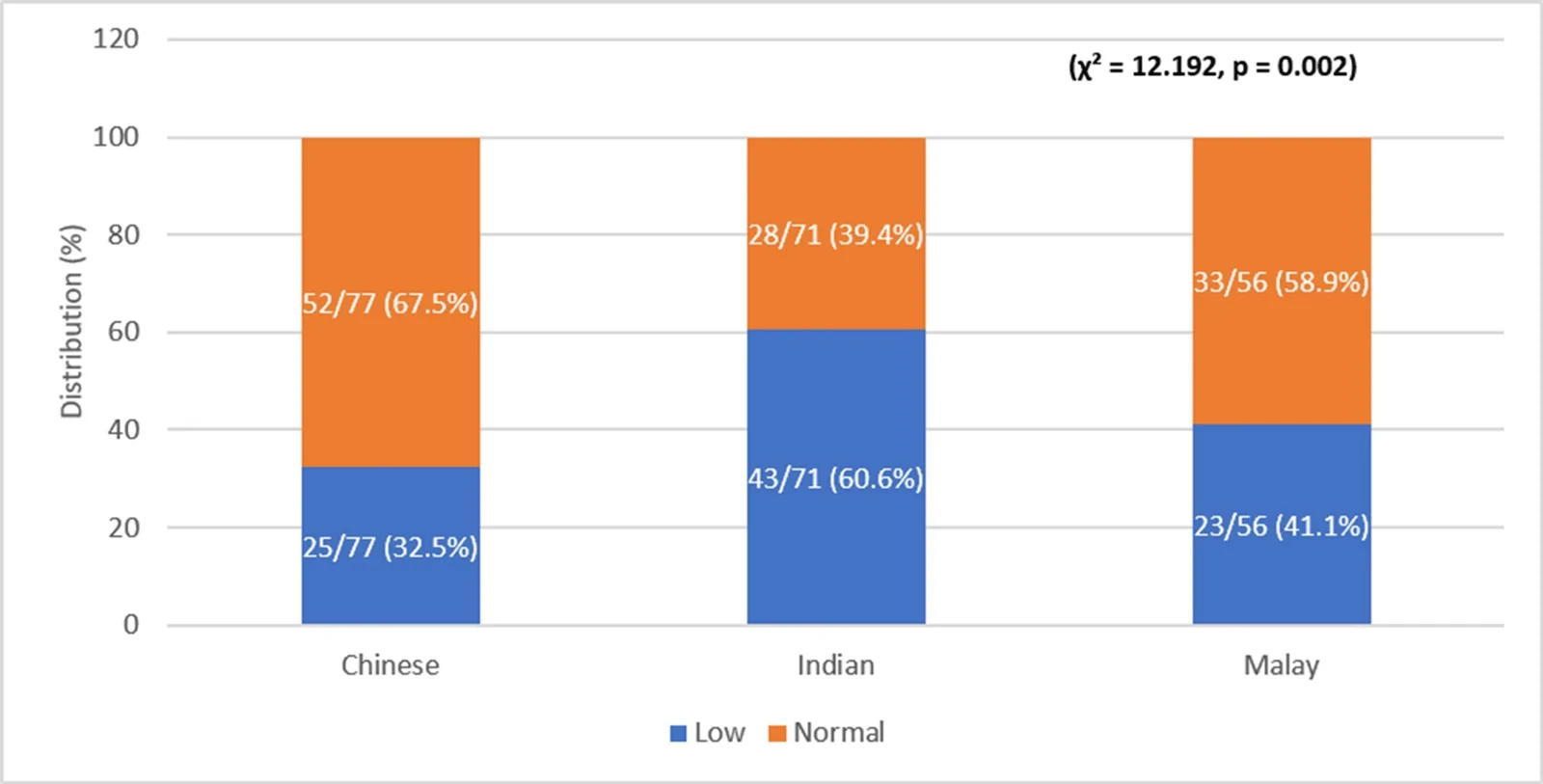
Serum albumin levels within each ethnic group. χ² = chi-square test

## Discussion

BMD and T-score discordance

In this cohort, 28.9% (n = 59) of patients were osteoporotic, and a further 59.3% (n = 121) were osteopenic. BMD assessed by DXA remains the gold standard for the diagnosis of osteoporosis, with the diagnostic threshold set at a T-score of ≤ -2.5. It is most commonly assessed at the lumbar spine and proximal femur. BMD is also used to monitor the response to antiresorptive or anabolic therapy. It is not, however, without limitations.

T-scores at the lumbar spine (L1-L4) carry a risk of being falsely elevated, as degenerative changes (sclerosis) are common in individuals older than 65 years of age. On the other hand, the lumbar region remains a sensitive reflection of postmenopausal bone loss, since oestrogen deficiency predominantly affects the trabecular bone of the spine [10].

The hip comprises both cortical and trabecular bone. Hip BMD correlates most closely with hip fracture risk, which in turn carries substantial morbidity and mortality; however, inadequate internal rotation of the femur during positioning can result in a falsely elevated score.

The distal forearm is predominantly composed of cortical bone. In patients with hyperparathyroidism, in whom cortical bone is greatly affected, this site most accurately reflects current bone status. The distal forearm also serves as a useful alternative site when the spine or hip cannot be assessed (e.g., due to prior implants). It is, however, unsuitable for monitoring the response to anti-osteoporotic treatment, as cortical bone remodels more slowly than trabecular bone.

Trabecular bone score (TBS) is an adjunct to DXA that estimates bone microarchitecture from the degree of grey-level variation among pixels on lumbar spine images. Combined with DXA, TBS improves fracture risk stratification and refines the FRAX score; it has been shown to reclassify up to 30% of patients from a ‘safe’ category to a higher fracture-risk category [11].

Radiofrequency echographic multi-spectrometry (REMS) is an ultrasound-based, radiation-free, and portable modality, although its accuracy is operator-dependent and sonographic penetration is limited by the overlying soft-tissue envelope. A systematic review reported a reliable correlation between REMS- and DXA-derived BMD, with the advantage of being unaffected by degenerative spinal sclerosis [12]. REMS also generates a Fragility Score derived from bone microarchitecture.

In our study, no significant association was found between BMD and either calcium or vitamin D levels. This finding is not unexpected, as BMD is influenced by multiple additional factors, including comorbidities, medication use, lifestyle, diet, and sex.

The relevance of correcting calcium

Although serum albumin levels were measured initially to calculate corrected serum calcium levels, they were ultimately not used in the final analysis. This cohort comprised elderly and potentially malnourished patients, and calcium correction in such patients may tend to overestimate true calcium status, potentially obscuring those with hypocalcaemia and increasing the apparent number of patients classified as hypercalcaemic [13]. In this study, 6.4% (n = 13) of patients had hypercalcaemia using unadjusted total calcium, and this proportion rose to 22.1% (n = 45) when Payne's formula was applied.

This observation is consistent with a large 2025 study of over 22,000 patients, in which unadjusted total calcium showed substantially better agreement with true ionised calcium status (74.5%) than the standard Payne correction formula (58.7-63.0%) [14].

Vitamin D: levels and significance

Vitamin D, a hormone, is most commonly associated with skeletal health, but its physiological role is considerably broader; vitamin D receptors are expressed in the brain, heart, skeletal muscle, and immune cells. Within skeletal metabolism, its principal role is to promote intestinal calcium absorption, which is estimated to account for approximately 10-15% of dietary calcium absorption. Vitamin D acts together with parathyroid hormone (PTH) to maintain bone health: PTH secretion increases when vitamin D levels fall below 30-50 nmol/L (deficiency), helping to maintain serum calcium through increased bone resorption. This process, however, increases the risk of fracture.

Vitamin D also supports immune function, promoting the synthesis of cathelicidins and other antimicrobial peptides by macrophages and monocytes. Vitamin D receptors are expressed on type II (fast-twitch) skeletal muscle fibres, which are central to postural balance and rapid, powerful movements. This may confer a protective effect against falls and fractures in older adults, who are likely to be vitamin D insufficient. Sufficient vitamin D status has been associated with faster postoperative recovery, reduced infection risk, and better bone health [1,2].

The prevalence of vitamin D deficiency in our study (43.6%) was comparable to that reported among Europeans (40%), Canadians (37%), and the global population (47.9%), as reported in a meta-analysis by Cui et al. in 2023 [15,16]. In contrast, some neighbouring countries have lower prevalence of vitamin D deficiency, with rates of 6% in Thailand and 13% in Singapore [17]. It is possible that the lower prevalence of vitamin D deficiency reported among Singaporean and Thai populations may partly reflect differences in population composition, including the proportion of individuals of Chinese ancestry. In our study, a clear ethnic gradient in vitamin D deficiency was observed (Malay > Indian > Chinese). These differences are likely due to differences in sun exposure, nutritional awareness and dietary intake, skin pigmentation (as melanin reduces cutaneous vitamin D synthesis), and clothing or cultural practices.

Interestingly, serum vitamin D thresholds are not uniformly defined. The Institute of Medicine, the Endocrine Society, and the American Association of Clinical Endocrinologists each specify different cut-offs for defining deficiency, insufficiency, and sufficiency. In this study, in keeping with Malaysian clinical practice, the Endocrine Society thresholds were applied.

There are limitations to this study. The cohort in this study was predominantly composed of patients aged over 60 years, and the sample size was relatively small. This study did not measure the various factors that could affect serum vitamin D levels and bone health, nor did it assess the potential postoperative impact on this cohort of patients undergoing arthroplasty surgery. However, it provides a foundation for further studies in this area and may inform future clinical practice.

## Conclusions

In this cohort of elderly Malaysian cohort scheduled for elective arthroplasty, the prevalence of vitamin D deficiency and osteoporosis was high. However, vitamin D deficiency did not translate into lower BMD, consistent with the multifactorial determinants of bone density. These findings underscore the high burden of modifiable metabolic bone risk factors in patients presenting for elective arthroplasty in Malaysia and support the routine incorporation of vitamin D, BMD, and nutritional screening into preoperative BHO pathways.
